# A case of gabapentin overdose induced rhabdomyolysis requiring renal replacement therapy

**DOI:** 10.1002/ccr3.2302

**Published:** 2019-07-11

**Authors:** Xiaoliang Qiu, Eva Tackett, Zeid Khitan

**Affiliations:** ^1^ Department of Medicine Marshall University Joan C. Edwards School of Medicine Huntington West Virginia

**Keywords:** acute kidney injury, gabapentin, hemodialysis, rhabdomyolysis

## Abstract

We report a rare case with gabapentin overdose that caused severe rhabdomyolysis and acute tubular necrosis which required renal replacement therapy. A better awareness of its adverse effect and a close follow‐up of laboratory tests are recommended. Prescribers should also be aware of high‐risk population and monitor for signs of abuse.

## 1 INTRODUCTION

Gabapentin is an anticonvulsant drug, also used in neuropathic pain caused by herpes zoster or diabetes, and restless legs syndrome. It has more and more widely used for off‐label indications such as migraine, fibromyalgia, mental illness, and substance dependence.^1^ Its common side effects include dizziness, drowsiness, ataxia, fatigue, tremor, nausea, and vomiting. Here, we report a rare toxicity by gabapentin overdose: rhabdomyolysis and acute renal failure. To date, only five cases reported gabapentin‐induced rhabdomyolysis in PubMed database.

## 2 CASE REPORT

We report a 39‐year‐old male patient presented to hospital with altered mental status and acute kidney injury secondary to rhabdomyolysis most likely due to gabapentin overdose. He took 13 pills of 400‐mg gabapentin and ¼ strip of suboxone with an unclear dose at a friend's house. He was found in confusion and vomiting by paramedics, sent to a local hospital and then transferred to our hospital. Initial laboratory and follow‐up laboratory please refer to Table 1.

**Table 1:** Relevant laboratory results of the patient

	Day 1	Day 2	Day 3	Day 4	Day 5	Day 6	Day 7	Day 8	Day 9	Day 10	Day 11	Day 12	3 wk	3 mo
CK (IU/L)	52 800	41 490	14 010	4611	2601	1329	714	431	315	195	161	192	90	
LDH (IU/L)		1822	837	479	412	355								
Cr (mg/dL)	4.40	5.30	7.54	6.86	6.60	6.37	8.25	7.55	8.39	6.49	6.74	6.30	1.63	1.23
Serum myoglobin (ng/mL)	20 000			1239										

His past medical history includes hypothyroidism, hypertension, restless leg syndrome, anxiety/depression, hyperlipidemia, ureteral stenosis status post urethral dilation, and recovery of drug use. Patient has no history of renal disease. Past surgical history is cholecystectomy and urethral dilatation. For social history, he denied tobacco and alcohol use but did have history of prescribed drug abuse. He denied any intravenous drug use, any recent use of opioids and cocaine. Family history is significant for hypertension and coronary artery disease. His home medications include aspirin 81 mg daily, atenolol 12.5 mg twice a day, atorvastatin 40 mg daily, Flomax 0.4 mg daily, gabapentin 1200 mg at bedtime, omeprazole 40 mg daily, clonazepam 0.5 mg daily, mirtazapine 45 mg at bedtime, fluoxetine 20 mg daily, trazodone 200 mg at bedtime, and Synthroid 100 mcg daily.

On physical examination, vital signs were stable with temperature 98.2°F, heart rate 99 bpm, respiratory rate 15 breath per minute, blood pressure 142/76 mm Hg, O_2_ saturation 95% on 40% venturi mask, and then was tapered to room air. He had no acute distress. Other examinations were unremarkable except decreased breath sound on the left side compared with the right. Creatinine kinase (CK) was 52 800 IU/L at admission (see Table 1 for LDH, myoglobin, and CK trend). Arterial blood gas in outside facility revealed 7.21/53/54. Laboratory in outside facility: WBC 17, Hb 13.2, platelet 317, Sodium 132 mmol/L, Potassium 6.3 mmol/L, CO_2_ 21 mmol/L, BUN 26 mg/dL, Cr 3.38 mg/dL, Glucose 126 mg/dL, Calcium 8.2 mg/dL, AST 157 IU/L, ALT 61 IU/L, Lactic acid 5.3 mmol/L, Total Bilirubin 1.3 mg/dL, and alkaline phosphatase 78 IU/L. Urine drug screen was positive for buprenorphine and benzodiazepine. Urinalysis revealed small amount leukoesterase, mild white blood cell, and trace bacteria. Blood culture and urine culture were negative. Chest X‐ray showed left side infiltrates. EKG was normal sinus rhythm without ischemic ST‐T changes. CT chest was consistent with pulmonary infiltrates with left worse than the right side. Ultrasound of right upper quadrant and kidney was normal. He was treated in ICU and was followed by nephrology. Patient received aggressive hydration and seven times hemodialysis. He also has aspiration pneumonia and was treated by unasyn and then by augmentin and finished 6‐day course. He had non‐ACS hypertroponinemia, troponin from 9 to 11, which was cleared by cardiology. He had no EKG changes, asymptomatic, with echocardiography EF > 55%.

## 3 DISCUSSION

Gabapentin functions as a γ‐aminobutyric acid (GABA)‐mimetic agent, binding to the alpha‐2‐delta subunit of the voltage‐gated calcium channels.^2^ Gabapentin was originally approved by the US Food and Drug Administration (FDA) in 1993 for epilepsy and later, postherpetic neuralgia.^1^ Recently, gabapentin has been increasingly used in some off‐label indications as a desired alternative of opioids for pain management,^1^, ^3^ probably due to its relative low rate of adverse effect and low cost. However, in recent years, reports of recreational gabapentin abuse or intentional misuse have increased at an alarming rate with reported adverse effects.^4^ The potential for abuse was reported to be particularly in individuals with a history of opioid abuse, and the adverse effect is more common when combined with opoids.^4^ This patient has a history of drug abuse and is on suboxone for treating opioid addiction. Therefore, this patient is at high risk for gabapentin adverse effect.

Gabapentin is highly lipid and water soluble and is not metabolized. Gabapentin is excreted by kidneys. Impaired renal function results in higher plasma gabapentin concentrations, longer elimination half‐lives, and reduced renal clearance.^5^ Clinically, it is advisable to adjust gabapentin dose per renal function, but it was often overlooked. Surprisingly, massive gabapentin overdose has been associated with few adverse effects.^6^, ^7^, ^8^, ^9^ Gabapentin toxicity has been reported in patients with chronic kidney disease or hemodialysis. In these cases, gabapentin was noted to cause tremors, altered mental status, and respiratory depression requiring intubation.^10^ Rhabdomyolysis is an extremely rare adverse effect of gabapentin. Our case of rhabdomyolysis caused by gabapentin overdose abuse is the first case to our knowledge.

Rhabdomyolysis is a clinical syndrome caused by dissolution of skeletal muscle and release of its breakdown products including electrolytes, myoglobin, and other sarcoplasmic proteins (eg, creatine kinase, aldolase, lactate dehydrogenase, alanine aminotransferase, and aspartate aminotransferase) into the circulation.^11^ Pathogenesis is not clear, but it appears to involve depletion of cytosolic ATP paralleled with calcium overload and thereby activation of calcium‐dependent proteases, which lead to destruction of myofibrils and lysosomal digestion of muscle fiber contents.^12^ Interestingly, cardiac tissue is hardly affected or only secondary, as a consequence of imbalance in electrolytes or acid‐base equilibrium.^13^ Clinically, it varies from asymptomatic to muscle weakness, myalgia, swelling, and gross myoglobinuria. Severe rhabdomyolysis can cause acute kidney injury which indicates a worse prognosis. Our patient has remarkably elevated CK, LDH, serum myoglobin, and creatinine which confirmed the diagnosis of rhabdomyolysis.

For differential diagnosis, trauma, exertion, genetic defects, muscle hypoxia, infection, body‐temperature changes, drugs and toxins, metabolic, and electrolyte disorders are common causes of rhabdomyolysis.^1^ Lipid‐lowering drugs (fibrates, statins), alcohol, heroin, and cocaine were common drugs that can cause rhabdomyolysis. For our patient, he denied any recent trauma, seizure, any intense physical activities, opioid and cocaine intake, and family history of muscular disease. He had no fever, ruling out fever‐induced rhabdomyolysis. Laboratory work ruled out severe abnormalities of electrolytes. Blood culture and urine culture did not reveal any infection. His home medications included atorvastatin, clonazepam, and omeprazole which he took for a long time, and he did not take other medications which were commonly associated with rhabdomyolysis. Furthermore, it was not clear exactly how long the patient had been unresponsive per patient's history. To our best knowledge, patient took gabapentin at the night and was found next morning, so it may be at least a few hours. Therefore, immobilization might be a contributing factor as well.

Gabapentin‐induced rhabdomyolysis is rare. Seven reported cases of gabapentin‐induced myopathy in PubMed database but only five cases are rhabdomyolysis, see Table2.^2^, ^14^, ^15^, ^16^, ^17^ Lipson et al reported two cases of myopathy in patients receiving hemodialysis developed neuromuscular symptoms and a lower level of elevation of creatine kinase after starting gabapentin therapy with longer symptoms onset (2 weeks and 6 weeks, respectively). Patient is already on hemodialysis, so it is less likely it is acute rhabdomyolysis per se. Five cases listed here had relatively acute onset of symptoms. Four of them required hemodialysis, peritoneal dialysis, or continuous renal replacement therapy. Symptoms and laboratory findings were resolved after gabapentin discontinuation. Of note, as aforementioned, gabapentin toxicity has been reported in patients with chronic kidney disease or dialysis. The case reported by Bilgir et al had CKD stage 3 and the Italian case by Falconi et al had chronic renal failure on peritoneal dialysis. It further confirmed gabapentin dose should be renally adjusted. In our case, patient did not have any renal disease before, but he did have drug use history and was on suboxone for treatment. Of interest, suboxone has not been reported to cause rhabdomyolysis so far. Patient was also on scheduled benzodiazepine clonazepam and atorvastatin which are reported to be associated with rhabdomyolysis.^13^, ^18^ However, due to the acute onset, it is less likely clonazepam and atorvastatin caused the rhabdomyolysis.

**Table 2:** A summary of reported five cases of rhabdomyolysis due to gabapentin therapy

Year	Author	Age	Sex	Dose	Duration of gabapentin	Indication	Other medications	Treatment
2007	Tuccori et al^17^	85	F	150 mg TID	1 d	Diabetic neuropathy	Insulin, ramipril, aspirin, diltiazem, haloperidol, lansoprazole	Conservative
2009	Bilgir et al^14^	63	F	300 mg TID	3 wk	Diabetic neuropathy	Insulin, irbesartan	HD
2012	Torregrosa et al^16^	49	M	600 mg q8h	48 h	Low back pain	Eprosartan, bisoprolol, paroxetine, mianserin, disulfiram	HD
2015	Folconi et al^15^	65	M	NA	3 d	Diabetic neuropathy	NA	CAPD dialysis
2017	Choi et al^2^	32	F	600 mg TID	1 mo	Low back pain	Hydromorphone	CRRT

The patient's CK, LDH, and myoglobin got resolved after the initiation of hemodialysis (Table 1). His urine output got improved in 24 hours. His AKI is most likely due to acute tubular necrosis secondary to rhabdomyolysis. His Cr peaked at 8.39 on day nine and trended down slowly. He was followed up in about 3 weeks after the event, and his Cr was 1.63. Two months later, his Cr was 1.23 and no hematuria and proteinuria were detected. He regularly followed up with nephrology with stable kidney function.

In conclusion, we report a case with gabapentin overdose that caused severe rhabdomyolysis and AKI likely due to acute tubular necrosis. Renal replacement therapy should be initiated in severe cases. Since gabapentin toxicity in patients with chronic kidney disease is often overlooked, a better awareness of its adverse effect and a close follow‐up of laboratory tests is suggested.^15^ Appropriate dosing adjustments should be made in patients receiving gabapentin in case of renal insufficiency. On the hand, our case reinforced the policy that gabapentin be the schedule V controlled substances in more and more states.^3^ Prescribers should be aware of high‐risk population and monitor for signs of abuse.

## CONFLICT OF INTEREST

None declared.

## AUTHOR CONTRIBUTIONS

Data collection: XQ and ZK. Literature search and wrote the paper: XQ. Review the paper: ZK and ET.
